# Correction: Ramírez-Uclés et al. Validation and Spanish Adaptation of the Resilience Scale ER-23 in a University Population. *Healthcare* 2025, *13*, 886

**DOI:** 10.3390/healthcare13111259

**Published:** 2025-05-27

**Authors:** Isabel Ramírez-Uclés, Julia Otero, F. Pablo Holgado-Tello, Lucas Muñoz-López, María B. Sánchez-Barrera

**Affiliations:** 1 Department of Personality, Assessment and Psychological Treatment, Universidad Nacional de Educación a Distancia (UNED), 28040 Madrid, Spain; 2 Brain, Main and Behaviour Research Center, University of Granada, 18071 Granada, Spain; juliaotero@ugr.es (J.O.); lucasml@ugr.es (L.M.-L.); mbsanch@ugr.es (M.B.S.-B.); 3 Department of Behavioral Sciences Methodology, Universidad Nacional de Educación a Distancia (UNED), 28040 Madrid, Spain; pfholgado@psi.uned.es; 4 Department of Personality, Assessment and Psychological Treatment, University of Granada, 18071 Granada, Spain

With this Correction, the authors would like to replace references 46, 48 and 50 in the published paper [1] with the following new ones:

46. Guillén-Riquelme, A.; Buela-Casal, G. Actualización psicométrica y funcionamiento diferencial de los ítems en el State Trait Anxiety Inventory (STAI) [Psychometric update and differential item functioning in the State Trait Anxiety Inventory (STAI)]. *Psicothema* **2011**, *23*, 510–515.
48. Holgado-Tello, F.P.; Chacón–Moscoso, S.; Barbero-García, I.; Vila–Abad, E. Polychoric versus Pearson correlations in exploratory and confirmatory factor analysis of ordinal variables. *Qual. Quant.* **2010**, *44*, 153–166.
50. Yang-Wallentin, F.; Jöreskog, K.G.; Luo, H. Confirmatory factor analysis of ordinal variables with misspecified models. *Struct. Equ. Model.* **2010**, *17*, 392–423.

The authors state that the scientific conclusions are unaffected. This correction was approved by the Academic Editor. The original publication has also been updated.
